# Pelvic lymphadenectomy in vulvar cancer and its impact on prognosis and outcome

**DOI:** 10.1007/s00404-021-06156-x

**Published:** 2021-08-13

**Authors:** A. Jaeger, K. Prieske, S. Mathey, I. Fischer, E. Vettorazzi, S. Kuerti, S. Reuter, J. Dieckmann, B. Schmalfeldt, L. Woelber

**Affiliations:** 1 Department of Gynecology and Gynecologic Oncology, University Medical Center Hamburg-Eppendorf, Martinistraße 52, 20246 Hamburg, Germany; 2 Mildred Scheel Cancer Career Center HaTriCS4, University Medical Center Hamburg-Eppendorf, Hamburg, Germany; 3 Department of Medical Biometry and Epidemiology, University Medical Center Hamburg-Eppendorf, Hamburg, Germany

**Keywords:** Vulvar cancer, Groin, Lymphadenectomy, Pelvic nodal involvement, Prognosis

## Abstract

**Background:**

The value of pelvic lymphadenectomy (LAE) has been subject of discussions since the 1980s. This is mainly due to the fact that the relation between lymph node involvement of the groin and pelvis is poorly understood and therewith the need for pelvic treatment in general.

**Patients and Methods:**

*N* = 514 patients with primary vulvar squamous cell cancer (VSCC) FIGO stage ≥ IB were treated at the University Medical Center Hamburg-Eppendorf between 1996 and 2018. In this analysis, patients with pelvic LAE (*n* = 21) were analyzed with regard to prognosis and the relation of groin and pelvic lymph node involvement.

**Results:**

The majority had T1b/T2 tumors (*n* = 15, 78.9%) with a median diameter of 40 mm (11–110 mm). 17/21 patients showed positive inguinal nodes. Pelvic nodal involvement without groin metastases was not observed. 6/17 node-positive patients with positive groin nodes also had pelvic nodal metastases (35.3%; median number of affected pelvic nodes 2.5 (1–8)). These 6 patients were highly node positive with median 4.5 (2–9) affected groin nodes. With regard to the metastatic spread between groins and pelvis, no contralateral spread was observed. Five recurrences were observed after a median follow-up of 33.5 months. No pelvic recurrences were observed in the pelvic nodal positive group. Patients with pelvic metastasis at first diagnosis had a median progression-free survival of only 9.9 months and overall-survival of 31.1 months.

**Conclusion:**

A relevant risk for pelvic nodal involvement only seems to be present in highly node-positive disease, therefore pelvic staging (and radiotherapy) is probably unnecessary in the majority of patients with node-positive VSCC.

## Introduction

Within the last two decades, the incidence of vulvar squamous cell cancer (VSCC) constantly increased and eventually doubled to currently 3–5/100,000/year in Europe [1]—nonetheless, VSCC remains a rare disease comprising approximately 5–6% of all gynecological malignancies [2]. Lymph node involvement is known to be the most important factor in terms of prognosis and outcome as represented in 3-year PFS rates of 35.2% and OS rates of 56.2% in node-positive patients vs. 3-year PFS rates of 75.2% and OS rates 90.2% in node negative disease [3–8]. In general, pelvic nodal involvement is estimated to occur in less than 10% of all VSCC, but in 20–35% of node-positive VSCC [3, 9–11]. However, established clinical data concerning the pelvic spread, its impact on prognosis and outcome as well as information regarding the correlation of inguinal and pelvic nodal involvement are lacking, and so are evidence if and when to perform pelvic LAE and/or radiation of the pelvis at all. The number of groin nodes affected seems to correlate with the risk of experiencing pelvic metastases—in this context, an increasing number of metastatic groin nodes is associated with an elevated risk for pelvic nodal involvement [12, 13]. Nonetheless, valid prediction of pelvic nodal involvement based on clinical or imaging—procedures remains an unresolved issue.

As a consequence, the population at risk for pelvic nodal involvement remains insufficiently understood and the benefit of pelvic treatment endures to be an unanswered question until today. In order to prevent unnecessary harm in form of substantial morbidity caused by radiotherapy of the pelvis, the German Guidelines recommend to perform pelvic LAE as a staging procedure only in patients at risk for pelvic nodal involvement [6]. On the one hand, this therapeutic approach may prevent morbidity mostly caused by radiotherapy, on the other hand, pelvic LAE as a staging procedure often requires a second surgery and eventually comes along with increased, surgery-related morbidity. Irrespective thereof, the question when and how to perform pelvic LAE as well as the extent of pelvic treatment in general in patients with node-positive VSCC has been und still is surrounded by considerable controversy. Therefore, the aim of this study was to investigate the relation between inguinal und pelvic lymph node involvement (Table 1).

**Table 1:** Patients characteristics (*n* = 21) with regard to pelvic lymph node status

Characteristics	Missing	Pelvic *N* + * n* = 6	Pelvic *N*−*n* = 15	Total * n* = 21	*p* value
Age median (range)	0	56.5 (37.0–70.0)	53.0 (28.0–71.0)	53.0 (28.0–71.0)	0.668^a^
Tumor stage	2				
p T1b		3 (60%)	7 (50%)	10 (52.6%)	
p T2		1 (20%)	4 (28.6%)	5 (26.3%)	
p T3/4		1 (20%)	3 (21.4%)	4 (21.1%)	
Nodal status (groin)					
N 0		0 (0%)	4 (26.7%)	4 (19.1%)	0.281^2^
N 1		6 (100%)	11 (73.3%)	17(80.9%)	
Number of groin nodes affected median (range)	2	4.5 (2.0–9.0)	1 (0.0–5.0)	2.0 (0.0–9.0)	0.010^a^
Max diameter LN met groin (range)	5	45.0 mm (23–54)	18.0 mm (1–40)	23.0 mm (1–54)	0.027^a^
Number of pelvic nodes affected median (range)		2.5 (1–8)	0.0	0.0 (0–8)	< 0.001^a^
Depth of invasion mm median (range)	6	11.5 (7–16)	6.0 (1–35)	7.0 (1–35)	0.349^a^
Grading					
G 1		0 (0%)	0 (0%)	0 (0%)	
G 2		4 (66.7%)	5 (33.3%)	9 (42.9%)	
G 3		2 (33.3%)	8 (53.3%)	10 (47.6%)	
Unknown		0	2 (13.4%)	2 (9.5%)	
Surgical therapy vulva					
Wide excision		2 (33.3%)	1 (6.7%)	3 (14.3%)	
Partial vulvectomy		1 (16.7%)	8 (53.3%)	9 (42.8%)	
Complete vulvectomy		1 (16.7%)	4 (26.6%)	5 (23.8%)	
No surgical treatment/unknown		2 (33.3%)	2 (13.3%)	4 (19.1%)	
Resection margin mm median (range)	11	2.4 (0.9–4.0)	3 (0.0–6.0)	3 (0.0–6.0)	0.595^a^
Resection status	3				
R 0		3 (60%)	8 (61.5%)	11 (61.1%)	
R 1		1 (20%)	4 (30.8%)	5 (27.8%)	
R x		1 (20%)	1 (7.7%)	2 (11.5%)	
Groin dissection	1				
Unilateral		1 (16.7%)	2 (14.3%)	3 (15.0%)	1.000^2^
Bilateral		5 (83.3%)	12 (85.7%)	17 (85.0%)	
Pelvic LN dissection					
Unilateral		2 (33.3%)	4 (26.7%)	6 (28.6%)	1.000^2^
Bilateral		4 (66.7%)	11 (73.3%)	15 (71,4%)	
Number of dissected groin LNs per patient median (range)		19 (12–24)	17 (6–27)	18 (15–38)	
Number of dissected pelvic LNs per patient median (range)		16 (6–27)	13 (1–42)	13 (1–42)	0.785^a^
Adjuvant therapy					
Radiotherapy only		1 (16.7%)	5 (33.3%)	6 (28.6%)	
Radiochemotherapy (RCTX)		4 (66.7%)	7 (46.7%)	11 (52.4%)	
Neoadjuvant RCTX		1 (16.7%)	2 (13.3%)	3 (14.3%)	
Radiation fields	1				
Groins ± vulva		0 (0%)	7 (50.0%)	7 (35.0%)	
Groins and pelvis ± vulva		6 (100%)	2 (14.3%)	8 (40%)	
Pelvis ± vulva		0 (0%)	0 (0%)	0 (0%)	
Vulva only		0 (0%)	1 (7.1%)	1 (5.0%)	
Groins only		0 (0%)	3 (21.4%)	3 (15.0%)	
Other (inguinal and pelvic)		0 (0%)	1 (7.1%)	1 (5.0%)	
Median PFS (months)	1	9.9	32.2	25.9	0.774^c^
Median OS (months)	1	31.1	112.2	32.2	0.398^c^

^a^Kruskal–Wallis rank sum test

^b^Fisher’s exact test for count data

^c^Survdiff logrank

## Methods

This retrospective subgroup analysis focuses on patients who were diagnosed with primary VSCC FIGO stage IB and higher and were treated with pelvic LAE (*n* = 21) at the University Medical Center Hamburg-Eppendorf between 1996 and 2018. Furthermore, the occurrence of pelvic nodal involvement at primary diagnosis as well as the correlation between inguinal and pelvic nodal involvement was evaluated. Also, the impact of pelvic nodal metastases on prognosis was investigated. Therefore, medical charts and pathological reports were reviewed. Previously, informed consent had been obtained from all included patients according to our investigational review board and ethics committee guidelines (Ethics Committee of the Medical Board Hamburg Reference Number 190504). Data collection were performed retrospectively between September 2019 and May 2020. Documentation and analysis were accomplished with the support of a Microsoft Excel database (2019). The approach to pelvic LAE in VSCC was heterogeneous in the investigational period as not recommended as a standard procedure in the guidelines at that point. Indication criteria were enlarged/suspicious pelvic nodes on radiologic exam or staging procedures to spare radiotherapy of the pelvis, e.g. in women with unfulfilled family planning. Furthermore the metastatic/nonmetastatic LN ratio (LN-R) was evaluated for pts with pelvic LAE (*n* = 21), see Tables 2 and 3. In 15/21 pelvic LAE was performed bilaterally, in 6 pts unilaterally (30 + 6 = 36 groins). According to Polterauer et al. [14] we defined LN-R as ratio of number of positive lymph nodes (LN) to the number of resected nodes. However, we calculated the number groin related not pts related in line with our previous analysis. Groins were stratified into three groups according to LN-R as previously described: intermediate risk group a LN-R of > 0% and < 20%, high risk group a LN-R of > 20%.

**Table 2:** Relation between inguinal und pelvic nodal involvement (*n* = 21 patients with pelvic LAE; *n* = 17 with known number of affected groin nodes, *n* = 6 with known pelvic metastases) and LN-R (lymph node ratio) for patients with positive pelvic LN

Pt (pelvic LAE u = unilateral, b = bilateral	Groin	No of lymph nodes resected	No of positive ( +) lymph nodes groin	Lymph node ratio	No of positive ( +) lymph nodes pelvis	No of patients with positive ( +) pelvic LN status (* n* = 6)
2 (b)	left	9	4	44.4%	2	x
2	right	3	1	33.3%	1	
5 (u)	left	18	9	50%	1	x
**8 (b)**	**left**	**8**	**1**	**12.5%**	**4**	**x**
**8**	**right**	**10**	**3**	**30%**	**4**	
13 (b)	left	–	0	–	0	
16 (u)	**left**	**10**	**2**	**20%**	**2**	**x**
17 (b)	**left**	**12**	**5**	**41.6%**	**3**	**x**
17	**right**	**–**	**1**	**–**	**0**

**Table 3:** LN—R for patients with pelvic LAE (*n* = 21). In 15/21 pelvic LAE was performed bilaterally, in 6 patients unilaterally (30 + 6 = 36 groins)

LN-R	Pelv−(*n* = 28)	Pelv + (*n* = 8)	Total (*n* = 36)	*p* value
N-Miss	3	0	3	
Mean (SD)	11.2 (13.9)	33.7 (12.6)	16.7 (16.6)	
Median (Range)	7.7. (0.0; 44.4)	35.4 (12.5, 50.0)	12.5 (0.0, 50.0)	

### Statistical analysis

Analysis was performed using Stata (StataCorp LP, Version 14.2). Variables are described as median and range or count and percentage, respectively. Receiver Operating Characteristics (ROC) analysis was performed and the area under the curve (AUC) was calculated to evaluate different cut-offs for the prediction of pelvic nodal involvement related to the number of affected groin nodes and the lymph node ratio (LN-R). Progression-free survival (PFS) was calculated as the time interval between primary diagnosis and disease progression or death of any cause, and overall survival (OS) was the period resulting from primary diagnosis to death of any cause. Univariate Cox regression analysis was applied to determine significant differences at a level of 5%. In accordance with the journal’s guidelines, we will provide our data for the reproducibility of this study in other centers if such is requested.

## Results

### Patients

Out of 514 patients with FIGO stage IB-IV (UICC-TNM-classification and stage-groupings version 6) [15] VSCC who were treated at the University Medical Center Hamburg-Eppendorf between 1996 and 2018, only 21 patients received pelvic LAE and had a known lymph node status of the groin. Patient characteristics are summarized in Table 1. Median age was 53 years (range: 28–71 years), median follow-up was 33.5 (range 3–120) months. The majority had locally restricted tumors (T1b/T2; TNM staging system Version 6 15/19; 78.9%) with a median diameter of 40 mm (11–110 mm) [15]. 17/21 (81%) patients with pelvic LAE showed positive inguinal nodes (N +) and 6/17 inguinal node-positive patients also had pelvic nodal metastases (35.3%) with a median number of 2.5 [1–8] affected pelvic nodes. These six patients were highly node positive with median 4.5 [2–9] affected groin nodes and a median metastatic diameter of 45.0 mm (23–54) in the groin. Pelvic nodal involvement without groin metastases did not occur. Furthermore, no contralateral dissemination, but a continuously side-consistent spread between groin and pelvis was observed within the total cohort. Bilateral pelvic LAE was performed in 15/21 (71.4%) patients—thereof 2/15 patients (13.3%) showed unilateral inguinal involvement with ipsilateral pelvic metastasis whereas another 2/15 patients (13.3%) experienced inguinal as well as pelvic metastasis on both sides. Table 2 highlights the correlation between inguinal und pelvic nodal involvement including the LN-R in these cases. In the current study, the positive predictive value for pelvic involvement in patients with ≥ 3 ipsilaterally affected lymph nodes in the groin was 62.5% while the negative predictive value was 88.5%. Adjuvant treatment was applied in 83.3% (5/6 pelvic nodal positive patients), 4 of these patients received chemoradiation. Furthermore, the LN-R was evaluated. With regard to the pelvic node-positive cohort, 87.5% (7/8 groins) were categorized into the high risk group (≥ 20%). Median LN-R within the pelvic node-positive subgroup was 35.4% (range 12.5–50.0) compared to 7.7% (0.0–44.4) in the pelvic node negative cohort (ROC Analysis showed an AUC of 0.88 for the prediction of pelvic involvement.
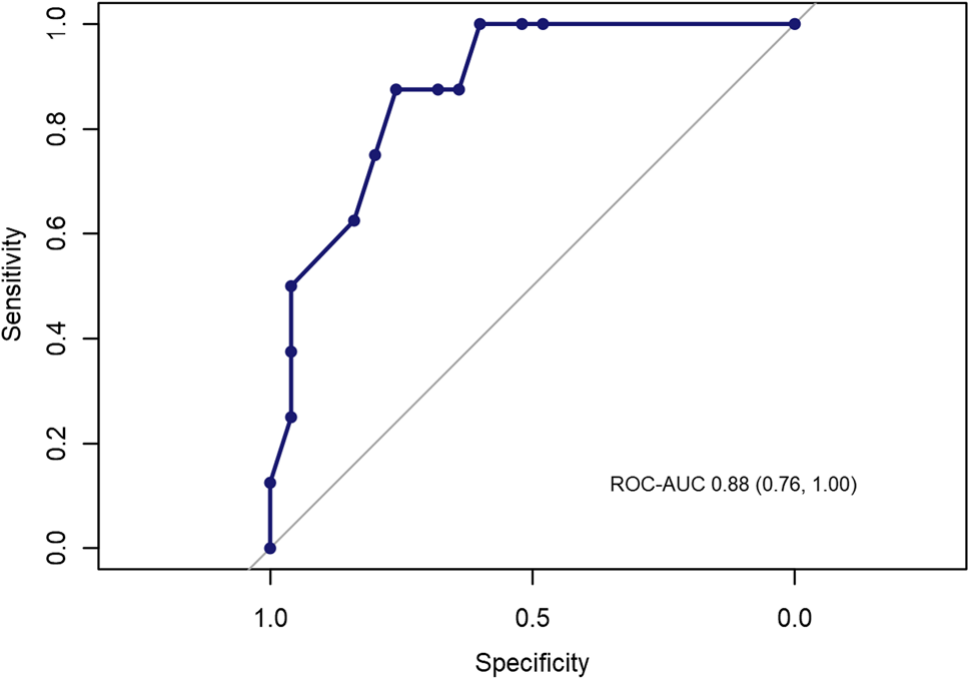


Evaluation of the prevalence of suspected pelvic lymph node involvement at first diagnosis within the entire data base (*n* = 514) revealed (only) two more patients with suspected positive pelvic lymph nodes (by imaging). So in the total cohort, 8 pts had (suspected) positive LN in the groin and the pelvis, thereof 6 have been included in our analysis and were treated with lymph node staging of the pelvis.

### Recurrences

A total of 23.8% (5/21) patients suffered from any kind of disease recurrence after a median follow up of 33.5 months (range 3–112 months) (Table 4). No pelvic recurrences were observed; neither in the pelvic node-positive group nor in the pelvic node negative cohort. In the pelvic node-positive group 33.3% (2/6) patients experienced distant recurrences as the most frequent site whereas in the pelvic node negative group, recurrences appeared most often at the vulva only (2/15 patients; 13.3%). As expected, the general risk of recurrence was higher in the pelvic node-positive group compared to the pelvic node negative group (3/6 patients, 50% vs. 2/15 patients, 13.3%). Within the follow-up of 33.5 months 86.7% (13/15) of node negative patients vs. 3/6, 50% node-positive patients remained free of recurrences.

**Table 4:** Site of disease recurrence

Localization of disease recurrence	Total (*n* = 21 Pt.) 5 recurrences (23.8%)	*N*−pelvic (* n* = 15 Pt.) 2 recurrences (13.3%)	*N* + pelvic (* n* = 6 Pt.) 3 recurrences (50%)
No recurrence	16 (76.2%)	13 (86.7%)	3 (50%)
Vulva only	2 (9.5%)	2 (13.3%)	0
Groins only	1 (4.7%)	0	1 (16.7%)
Vulva + Groins	0	0	0
Pelvis (± other localizations)	0	0	0
Distant (± other localizations)	2 (9.5%)	0	2 (33.3%)

### Prognosis

The median PFS for all patients regardless of the pelvic node status was 25.9 months while the median OS was 32.2 months. In case of pelvic metastasis prognosis was impaired with a median PFS of only 9.9 months and a median OS of 31.1 months (HR 5.34, 95%CI 2.17,13.12, *p* value < 0.001).

## Discussion

In this study, 21 patients treated with pelvic LAE were evaluated to investigate the relation between inguinal und pelvic lymph node involvement. Our results revealed no pelvic nodal involvement without lymph node metastases in the groin in accordance to previous published study results [16, 17]. Furthermore, our data suggest no contralateral spread between groin and pelvis and no pelvic recurrences within the initially pelvic node-positive patients were observed. Additionally, this study confirms an estimated risk for pelvic metastasis of approx. 30–35% in patients with node-positive VSCC in line with earlier reported data (Table 5, [4, 18]). However, in most of the sparse studies pelvic nodal involvement was evaluated patient-related instead of groin/side-related. Although it requires increased documentation effort, groin/side-related analyzation allows a better understanding of pelvic spread.

**Table 5:** Previous studies regarding the prevalence of pelvic LAE in patients with VSCC

	No of included pts	Rate of pelvic involvement	No of affected groin nodes	Analysis per groin and corresponding hemipelvis (y/n)
Homesly et al. [4]	53	28% (15/53 node + pts)	n.a	n
Klemm et al. [3]	12	17% (2/12 node + pts)	1–7	y
Woelber et al. [18]	70	33% (14/42 node + pts)	1–30	n
Woelber et al. [18]	21	35% (6/17 node + pts)	1–8	y

As of today, evidence regarding the extent of optimal pelvic treatment in VSCC is extremely limited. In 1986, the Gynecological Oncology Group (GOG) performed a randomized trial (GOG37) to compare the outcome and efficacy of a radiotherapy to both groins and pelvis vs. pelvic LAE without adjuvant treatment in patients with histologically proven, positive groin nodes after radical vulvectomy and bilateral groin dissection [4]. Herein, the 2-year OS was superior in the “radiotherapy group” in comparison to the “pelvic LAE group” (68% vs. 54%) whereas the pelvic recurrence rate was higher in the “radiotherapy group” (6% vs. 2%). The groin recurrence rate was concerningly higher in the “pelvic LAE” group compared to the “radiation group” (23.6% vs. 5.1%). As a result, the study had to be closed prematurely in view of clinically relevant survival benefit for the “radiation group”. However, the omittance of adjuvant radiotherapy to the positive groin was probably the main reason for the unfavorable results within the “pelvic LAE” group. Based on these study results, practice changing treatment recommendations have been implemented, leading to a standardized adjuvant radiotherapy of the groins AND pelvis in patients with VSCC with > 1 lymph node metastasis in the groin from that time on. Even though the study was not designed to answer the question when to treat the pelvic nodes at all.

However, most named studies are likely to overestimate the percentage of pelvic nodal involvement due to a negative selection bias as the decision for pelvic LAE was often made on an individual basis and before the publication of the current treatment guidelines. Therefore a relative overestimation of pelvic involvement with regard to all node-positive patients cannot be excluded [19]. Nevertheless, this also indicates that approximately 70% of all node-positive patients do probably not need any kind of pelvic treatment and also no surgical staging.

As mentioned earlier, survival in particular depends on the number of groin nodes affected (88% 2-year OS for patients with only one positive groin node, 66% 2-year OS for patients with 2–3 positive groin nodes and 2-year OS for patients with ≥ 4 positive groin nodes 27%, p < 0.0001) [4, 7]. Of note, (in the subanalysis of the CaRE study as well as in the current analysis), nodal spread to the pelvis was predominantly observed in patients with ≥ 4 positive groin nodes with a (median) maximum diameter of the lymph node metastasis in the groin of > 40 mm [18]. Therefore, the risk for pelvic nodal involvement appears to be of increasing relevance in highly groin node-positive disease. In the CaRE-1 subanalysis, valid predication of pelvic involvement could be made in ≥ 6 positive groin nodes [18], other previous smaller studies reported an increased risk for nodal spread to the pelvis in patients with ≥ 3 positive groin nodes [12, 20]. In this context, the LN-R as a possibly more accurate predictor for pelvic involvement has been assessed in our study. According to previous published study results, LN-R is an established prognostic parameter in a variety of solid tumors including cervical, endometrial, ovarian, and breast cancer [21]. Moreover, LN-R was able to predict survival more precisely than the number of positive LN and embodied an independent prognostic parameter in patients with node-positive VSCC within the VULCAN trial [22]. With regard to pelvic nodal involvement almost 90% of the patients (7/8 pts, 87.5%) with pelvic nodal involvement were in the high risk group with a LN-R > 20% while only one patient (1/8, 12.5%) was categorized in the intermediate risk group. As a result, LN-R turned out be a quite a good parameter, especially within the pelvic node-positive cohort, to potentially predict pelvic involvement. Another notable finding of our analysis was [12], that no pelvic recurrences occurred—neither within in the pelvic node negative nor within the pelvic node-positive cohort. Interestingly, results from the CaRE-1 subanalysis have proven slightly different outcomes in this particular case, e.g. a pelvic recurrences rate of 7% within the pelvic node negative patients compared to no pelvic recurrences in the node-positive subgroup [18]. Curry et al. previously observed a comparable pelvic recurrence rate of 8% in patients with negative pelvic nodes and < 4 positive groin nodes [13] while Homesley et al. detected a insignificantly lower pelvic recurrence rate of 4.4% (5/114 patients) within the total cohort and 1.8% (1/55 patients) within the “pelvic LAE” subgroup [23]. In this context, pelvic LAE might have been of beneficial impact or rather the omittance of pelvic radiotherapy in the pelvic node negative group might have increased the risk for pelvic recurrences. Furthermore, nodal spread to the pelvis is known to be closely linked to an extremely poor prognosis as represented in a PFS of 9.9 and an OS of only 31.1 months in pelvic node-positive patients. This raises further, clinically relevant, but still insufficiently answered questions:

1. *Are there any (and which) beneficial effects if positive nodes are found and removed?* Green et al. suggested that in up to 20% of all node negative cases submicroscopic disease is present and removed at the time of the surgery [16]—however, as for the available data, no study results have been presented to confirm or support these findings.
2. *Was the pelvic LAE unnecessary in case of histologically confirmed negative pelvic nodes (and in view of the poor prognosis)?* In this context, pelvic LAE may represents an alternative approach to avoid radiotherapy in a subset of patients at high risk for pelvic nodal involvement. This could especially be relevant for patients with comorbidities complicating radiotherapy or open family planning.

A further clinical problem is displayed by the fact that quite a considerable amount of inguinal node negative patients received pelvic LAE in our and other studies (19–23% [18]). Reactive enlargement of groin nodes clinically suspicious for metastatic disease probably serves as the most likely explanation for this issue. Hacker et al. previously observed that in up to 30% of clinical examinations, groin nodes have been declared as suspicious although final histology afterwards did not reveal any metastatic spread [12]. However, as a consequence, indication criteria should be taken under intensified reconsideration—particularly when given the fact that pelvic LAE adds nothing favorable to the cure of patients with histologically proven, negative pelvic lymph nodes as reported by Hacker et al. [12]. Even quite the contrary is the case: Pigge and Gaudenz reported that major complications were twice as common in patients having pelvic LAE than in those with groin dissection only [24]. In the light of these results, the role of radiologic imaging especially in early stage VSCC is about to continuously gain in importance to predict nodal involvement as precisely as possible. However, as of today, the accuracy of sonography ranges between 67 and 89% [25], sensitivity of MRI is 87% with an accuracy of 90% [26, 27] and sensitivity of PET is only 80% for detection of lymph node metastases [28]. Thus radiologic imaging is not quite ready to solve the problem of prediction of pelvic lymph node involvement in the majority of the cases (yet) [29]. As for the available data, simultaneous pelvic LAE without previous confirmation of groin metastases should therefore be omitted. The main restriction of our data is that the data were generated in times when pre-operative radiologic staging was not routinely implemented in all patients with locally advanced VSCC. Therefore, we missed to collect data and/or scans of radiologic imaging. We know the limitation of imaging regarding inguinal nodes, however, this is of subordinate relevance for the question we addressed in this work. In addition, due to the retrospective data documentation, a potential negative selection is most likely and has to be taken into account, too. Furthermore, extracapsular growth might also play a relevant role regarding the development of pelvic nodal involvement. However, this information was not collected in the AGO-CaRE-1 database, which can be considered as another limitation.

In conclusion, further systematic data collection including results of pre-operative imaging as planned by the German AGO study group, the AGO Kommission Vulva Vagina and the NOGGO are needed to clarify the indication criteria for pelvic LAE and/or radiotherapy as well as the impact of metastases on prognosis and outcome of affected patients.
